# Identification of Urinary Polyphenol Metabolite Patterns Associated with Polyphenol-Rich Food Intake in Adults from Four European Countries

**DOI:** 10.3390/nu9080796

**Published:** 2017-07-25

**Authors:** Hwayoung Noh, Heinz Freisling, Nada Assi, Raul Zamora-Ros, David Achaintre, Aurélie Affret, Francesca Mancini, Marie-Christine Boutron-Ruault, Anna Flögel, Heiner Boeing, Tilman Kühn, Ruth Schübel, Antonia Trichopoulou, Androniki Naska, Maria Kritikou, Domenico Palli, Valeria Pala, Rosario Tumino, Fulvio Ricceri, Maria Santucci de Magistris, Amanda Cross, Nadia Slimani, Augustin Scalbert, Pietro Ferrari

**Affiliations:** 1 Section of Nutrition and Metabolism, International Agency for Research on Cancer (IARC), 69372 Lyon CEDEX 08, France; NohH@fellows.iarc.fr (H.N.); AssiN@students.iarc.fr (N.A.); RaulZamoraros@gmail.com (R.Z.-R.); AchaintreD@iarc.fr (D.A.); SlimaniN@iarc.fr (N.S.); ScalbertA@iarc.fr (A.S.); FerrariP@iarc.fr (P.F.); 2 Unit of Nutrition and Cancer, Epidemiology Research Program, Catalan Institute of Oncology, Bellvitge Biomedical Research Institute (IDIBELL), L’Hospitalet de Llobregat, 08908 Barcelona, Spain; 3 Université Paris-Saclay, Université Paris-Sud, Université de Versailles Saint-Quentin-en-Yvelines (UVSQ), Le Centre de recherche en Epidémiologie et Santé des Population (CESP), Institut National de la Santé et de la Recherche Médicale (INSERM), 94800 Villejuif, France; Aurelie.AFFRET@gustaveroussy.fr (A.A.); Francesca.Mancini@gustaveroussy.fr (F.M.); Marie-christine.BOUTRON@gustaveroussy.fr (M.-C.B.-R.); 4 Gustave Roussy, 94800 Villejuif, France; 5 Department of Epidemiology, German Institute of Human Nutrition Potsdam-Rehbrücke, 14558 Nuthetal, Germany; Anna.Floegel@dife.de (A.F.); boeing@dife.de (H.B.); 6 Division of Cancer Epidemiology, German Cancer Research Center (DKFZ), 69120 Heidelberg, Germany; T.Kuehn@Dkfz-Heidelberg.de (T.K.); R.Schuebel@dkfz-heidelberg.de (R.S.); 7 Hellenic Health Foundation, 115 27 Athens, Greece; atrichopoulou@hhf-greece.gr (A.T.); anaska@hhf-greece.gr (A.N.); maria.kritikou@hhf-greece.gr (M.K.); 8 WHO Collaborating Center for Nutrition and Health, Unit of Nutritional Epidemiology and Nutrition in Public Health, Department of Hygiene, Epidemiology and Medical Statistics, University of Athens Medical School, 157 72 Athens, Greece; 9 Cancer Risk Factors and Life-Style Epidemiology Unit, Cancer Research and Prevention Institute (ISPO), 50139 Florence, Italy; d.palli@ispo.toscana.it; 10 Epidemiology and Prevention Unit, Department of Preventive and Predictive Medicine, Fondazione IRCCS Istituto Nazionale dei Tumori, 20133 Milano, Italy; Valeria.Pala@istitutotumori.mi.it; 11 Cancer Registry and Histopathology Unit, “Civic-M.P.Arezzo” Hospital, ASP Ragusa, 97100 Ragusa, Italy; rosario.tumino@asp.rg.it; 12 Unit of Epidemiology, Regional Health Service ASL TO3, 10095 Grugliasco (TO), Italy; fulvio.ricceri@unito.it; 13 Unit of Cancer Epidemiology, Department of Medical Sciences, University of Turin, 10124 Turin, Italy; 14 Azienda Ospedaliera Universitaria (AOU) Federico II, 80131 Naples, Italy; masantuc@unina.it; 15 Department of Epidemiology and Biostatistics, School of Public Health, Imperial College London, St Mary’s Campus, Norfolk Place, London W2 1PG, UK; amanda.cross@imperial.ac.uk

**Keywords:** dietary biomarker patterns, polyphenol metabolites, polyphenol-rich food, reduced rank regression (RRR), EPIC

## Abstract

We identified urinary polyphenol metabolite patterns by a novel algorithm that combines dimension reduction and variable selection methods to explain polyphenol-rich food intake, and compared their respective performance with that of single biomarkers in the European Prospective Investigation into Cancer and Nutrition (EPIC) study. The study included 475 adults from four European countries (Germany, France, Italy, and Greece). Dietary intakes were assessed with 24-h dietary recalls (24-HDR) and dietary questionnaires (DQ). Thirty-four polyphenols were measured by ultra-performance liquid chromatography–electrospray ionization-tandem mass spectrometry (UPLC-ESI-MS-MS) in 24-h urine. Reduced rank regression-based variable importance in projection (RRR-VIP) and least absolute shrinkage and selection operator (LASSO) methods were used to select polyphenol metabolites. Reduced rank regression (RRR) was then used to identify patterns in these metabolites, maximizing the explained variability in intake of pre-selected polyphenol-rich foods. The performance of RRR models was evaluated using internal cross-validation to control for over-optimistic findings from over-fitting. High performance was observed for explaining recent intake (24-HDR) of red wine (*r* = 0.65; AUC = 89.1%), coffee (*r* = 0.51; AUC = 89.1%), and olives (*r* = 0.35; AUC = 82.2%). These metabolite patterns performed better or equally well compared to single polyphenol biomarkers. Neither metabolite patterns nor single biomarkers performed well in explaining habitual intake (as reported in the DQ) of polyphenol-rich foods. This proposed strategy of biomarker pattern identification has the potential of expanding the currently still limited list of available dietary intake biomarkers.

## 1. Introduction

In nutritional epidemiology, the accurate and precise estimation of dietary exposures is critical for an unbiased assessment of diet–disease associations. Intakes of foods, nutrients or other bioactive compounds related to health or diseases are often estimated using self-reported dietary assessment methods, such as 24-h dietary recalls (24-HDR) or dietary questionnaires (DQs). However, the reliability of traditional self-reported instruments has been challenged due to inherent and sizeable measurement errors [1,2]. Dietary measurement errors are a serious challenge to establish reliable diet–disease associations [3].

Over the last decades, a limited number of dietary biomarkers have been identified and implemented in nutritional epidemiology [4,5]. They have been useful as reference measurements to validate self-reported dietary assessment tools (i.e., doubly labeled water and urinary nitrogen), as complementary measurements to compare with estimates of dietary intake (i.e., fatty acids, and carotenoids in blood), or as substitute measurements for insufficient or unavailable dietary intake data (i.e., selenium and zinc in blood) [4,6]. More recently, with the development of metabolomics, novel dietary biomarkers are being identified that should further improve the accuracy of dietary intake estimation [7,8].

These biomarkers have been mostly used individually for dietary exposure assessment. However, the ‘single biomarker’ approach has some conceptual and methodological limitations. First, single biomarkers cannot reflect complex matrices of dietary exposures with various food groups, which consist of multiple nutrients and other food components converted to a number of metabolites through various biological pathways, including the gut microbiota. Also, there are high inter-correlations among biomarkers, and some biomarker levels are too low to be detected or to reach a statistically significant performance for use in dietary intake assessment. Therefore, a ‘biomarker pattern’ approach may provide a more comprehensive and accurate measurement of complex dietary exposures. In this respect, some recent studies have used combinations of dietary biomarkers to improve the accuracy of dietary exposures assessment [9,10].

Polyphenols are non-nutritive plant components widely distributed in a variety of foods including fruits, vegetables, tea, coffee and wine [11]. Research interest in polyphenols has increased due to their potential protective effects on non-communicable diseases including cardiovascular diseases, diabetes and cancer, and premature mortality [12,13,14,15]. Overall results may be promising, but they remain inconclusive, and further prospective studies assessing dietary polyphenol exposure and studies using other methods to evaluate exposure (i.e., markers of consumption, metabolism, excretion) have been recommended, as concluded in a recent meta-analysis summarizing available evidence on the association of dietary flavonoid and lignan intake with cancer risk in observational studies [15]. Polyphenol metabolites measured in biological specimens could complement traditional dietary assessment tools to improve exposure assessment. A recent systematic review using intervention studies confirmed that urinary polyphenol metabolites could serve as dietary biomarkers with high recovery yields and high correlations with intakes of polyphenol-rich food [16]. Some single urinary polyphenol metabolites, such as, for example, chlorogenic acid/caffeic acid, gallic acid/resveratrol, caffeic acid/epicatechin, and naringenin/hesperetin have been identified as potential biomarkers for intakes of coffee, wine, tea, and citrus fruits/juices, respectively [17,18,19,20]. However, we hypothesized that panels or patterns of polyphenol metabolites may better explain intake of polyphenol-containing foods.

Recently, we reported correlations of 34 individual urinary polyphenol metabolites with intake of polyphenol-containing foods in the European Prospective Investigation into Cancer and Nutrition (EPIC) cross-sectional study [20]. Some single polyphenols were found to be significantly correlated to recent intake of these foods and were proposed as potential biomarkers of intake for these foods. In the current study, the same data were used to identify patterns of urinary polyphenol metabolites by applying a new algorithm that combines dimension reduction and variable selection methods to maximize the explained variation in intake of specific polyphenol-rich foods. The ability of these urinary polyphenol patterns to rank individuals according to the intake and to discriminate between consumers and non-consumers was examined and compared with the respective performance of single polyphenols.

## 2. Materials and Methods 

### 2.1. Subjects 

This study included 475 subjects randomly selected from four European countries (i.e., Germany, France, Italy, and Greece) within the EPIC calibration study, as described in our previous study [20]. In brief, the EPIC study is an ongoing multi-center prospective cohort study with more than half a million subjects, mostly aged 35–70 years, recruited from 23 centers in 10 European countries between 1992–2000. The study was designed to investigate relations between diet, lifestyle and environmental factors, and the risk of cancer and other chronic disease by collecting information on diet and lifestyle characteristics, anthropometric measurements, and medical history [21]. For the EPIC calibration study, a single 24-HDR was collected from a random sub-sample (*n* = 36,900) of the entire cohort, and a 24-h urine specimen was collected from a convenient sub-sample between 1995–1999 (*n* = 1386) of the calibration study [22,23]. For the current study, all subjects with available data in the form of a 24-h urine specimen and a 24-HDR collected on the same day, and a country-specific validated DQ collected at different time intervals (1 day–34 months) with regard to the 24-h urine collection across centers (*n* = 475), were eligible [24]. These subjects were recruited from the general population residing within defined geographical areas in Germany (Heidelberg and Potsdam), Greece (nationwide) and Italy (Naples, Turin, and Varese). Subjects had come to the study from breast cancer screening in Florence, a local blood donors association and their partners in Ragusa, Italy, and from an existing cohort. The latter was the case in France, where there was a cohort based on female teachers and school workers (Paris and surrounding areas). All subjects gave their informed consent for inclusion before they participated in the study. The study was conducted in accordance with the Declaration of Helsinki, and the protocol was approved by the ethical review boards of the International Agency for Research on Cancer (IARC) and from local participating institutions (Project identification code: doc. SC/24/6, date of approval: September 1987).

### 2.2. Dietary Assessment 

Dietary data were collected using a single standardized 24-HDR and a country-specific validated DQ. The 24-HDR face-to-face interview was conducted using a standardized dietary assessment methodology with a computerized program (EPIC-Soft) [22,25]. Dietary intake data using DQ with 158~266 items were self-administered or collected by face-to-face interviews to estimate usual intake over the previous 12 months [22].

### 2.3. Urinary Polyphenol Assessment

24-h urines were used for the measurement of urinary polyphenols. For the collection, subjects were provided two 2-L containers, each with 2 g boric acid as preservative. P-Aminobenzoic acid (PABA) was used as a marker for completeness of 24-h urine collections. After collection, 24-h urine samples were stored at −20 °C at the local center, and finally shipped within 24 h to and stored at −20 °C at the IARC, where laboratory analyses were performed after about 15 years of storage [19]. We do not expect major degradation of polyphenol metabolites during storage, and our previous studies using the same urine samples showed expected correlations between the metabolites and food intake [19,20]. As described previously [26], urine samples were first hydrolyzed with a β-glucuronidase/sulfatase enzyme mixture and the resulting polyphenol aglycones were extracted twice with ethyl acetate. Quantitative dansylation of phenolic hydroxyl groups was carried out with either 13C-dansyl chloride (samples) or non-labeled 12C-dansyl chloride (well-characterized reference pooled sample). Each 13C-dansylated sample was mixed with the 12C-dansylated reference sample, and the relative concentrations in samples over the reference were then measured by ultra-performance liquid chromatography–electrospray ionization-tandem mass spectrometry (UPLC-ESI-MS/MS). A total of 37 urinary polyphenols were measured, and their excretions in urine were expressed as μmol/24-h. Urinary polyphenol concentrations below the limit of quantification (LOQ) were replaced with values for half the LOQ. Since 98–100% of three polyphenols (procyanidins B1 and B2, and (+)-gallocatechin) values were below the LOQ, they were excluded from the analysis.

### 2.4. Statistical Analyses

Prior to the main statistical analyses, missing values of polyphenols were imputed by the expectation-maximization (EM) algorithm [27] after log transformation. In our previous study, center and batch were shown to explain a large part of the total variability of urinary polyphenols [20]. Urinary polyphenol measurements were therefore adjusted by taking the residuals from general linear models (GLMs), with center and batch variables as covariates. Intakes of food groups were log transformed and adjusted for energy intake by taking the residuals from GLMs with energy intake variable. Partial Pearson’s correlations between 34 individual polyphenols and the intakes of 12 main food groups and their 144 sub-groups (see Tables S1–S5) were computed conditional on sex, body mass index (BMI) and age as covariates.

An algorithm using dimension reduction and variable selection methods were applied to identify patterns of polyphenol metabolites and best explain the intake of polyphenol-rich foods. The procedure of the algorithm was as follows:

(1) Selecting optimal subsets of 34 polyphenol metabolites to explain intakes of specific polyphenol-rich food groups using two different variable selection methods: (i) variable importance in projection based on reduced rank regression (called the RRR-VIP method) [28] and (ii) least absolute shrinkage and selection operator (LASSO) regression [29].
(2) Identifying patterns of selected polyphenol metabolites (as predictor variables), and maximizing the explained variability of polyphenol-rich food group intakes (as response variables) through RRR analysis.
(3) Evaluating the performance of the RRR models for the polyphenol metabolite patterns to discriminate between consumers and non-consumers through internal two-fold cross-validation analyses. This was achieved through splitting the data into two equal-sized subsets (a training and a test set) and calculating (i) RRR scores in the test set using factor weights derived from RRR analysis of the training set; and (ii) Pearson correlation coefficients of RRR scores with intakes and area under the receiver operating characteristic curves (ROC AUCs) for the RRR scores of the test set.

For variable selection using the RRR-VIP method, a VIP score of each polyphenol metabolite, which is a weighted sum of squares of the RRR weights accounting for the explained variance of each RRR model, was calculated, and then polyphenol metabolites with a VIP score greater than 0.85 were selected [28,30]. Alternatively, we applied LASSO regression and its five-fold cross validation to select subsets of polyphenol metabolites by shrinking i.e., setting to 0, some coefficients of the predictors [29]. Partial Pearson correlation coefficients of RRR scores with intakes of polyphenol-rich foods from 24-HDR or DQ were calculated conditional on covariates (sex, BMI and age), and ROC AUCs were adjusted for these same covariates. All analyses were conducted using the Statistical Analysis Software, release 9.4 (SAS Institute Inc., Cary, NC, USA) and R software, version R.3.1.2 (R Foundation for Statistical Computing, Vienna, Austria).

## 3. Results

### 3.1. General Characteristics of the Study Population 

The average age and BMI of the participants were 54 ± 8.5 years and 26 ± 4.3 kg/m^2^, respectively. The percentage of smokers (former/current) and never smokers was 62% and 36% in men, and 36% and 61% in women, respectively. The proportion of subjects with prevalent diabetes, hyperlipidemia or hypertension was 2.5%, 27.2% and 23.6%, respectively (Table 1).

### 3.2. Correlations between Individual Polyphenol Metabolites and Polyphenol-Rich Food Groups 

In a first step, correlations between 34 individual polyphenol metabolites and the intakes of 12 main food groups and their 144 sub-groups in the EPIC study were explored to pre-select specific food groups that had sufficiently high correlations with a minimum set of polyphenol metabolites. Among the main food groups investigated, only four (‘vegetables’, ‘fruit, nuts & seeds’, ‘non-alcoholic beverages’, and ‘alcoholic beverages’) were significantly correlated with more than five individual polyphenol metabolites, while other main food groups were significantly correlated with less than three individual metabolites, and all coefficients were below 0.2 (Table S1). In a subsequent step, we examined correlations between polyphenol metabolites and food sub-groups (Tables S2–S5). Among these sub-groups, citrus fruits, apples and pears, olives, coffee, tea, all wine, and red wine were highly correlated with individual polyphenols (Table 2). For example, highly-correlated polyphenols were hesperetin (*r* = 0.54) and naringenin (*r* = 0.50) for citrus fruits, caffeic acid (*r* = 0.49) and ferulic acid (*r* = 0.42) for coffee, and gallic acid ethyl ester (*r* = 0.65) and resveratrol (*r* = 0.46) for red wine. All these food groups were selected for our multivariate analyses as polyphenol-rich food groups. The a priori arbitrarily defined criteria were that a given food group (or sub-group) showed a significant correlation with at least five polyphenols, and that at least one of these correlations was *r* ≥ 0.3. The criteria were chosen as a trade-off between having sufficiently informative predictor variables (i.e., polyphenols) and a wider range of potential food groups (i.e., response variables).

### 3.3. Selection of Polyphenol Metabolites Using Variable Selection Methods

Out of 34 urinary polyphenol metabolites, sub-sets were selected for identifying patterns associated with intakes of polyphenol-rich food groups using the RRR-VIP method and LASSO regression. Selected polyphenols differed by method, but at least the first one or two polyphenols were common in both methods (Table 3).

### 3.4. Identification of Polyphenol Metabolite Patterns Using Reduced Rank Regression

Patterns of selected polyphenol metabolites were identified through RRR analyses. Correlation coefficients and the AUC for the RRR scores of the polyphenol metabolite patterns were examined in the test set for cross-validation (Table 4). The RRR scores were highly correlated, with recent intakes of red wine (*r*_RRR-VIP_ = 0.65; *r*_LASSO_ = 0.66), citrus fruit (*r*_RRR-VIP_ = 0.54; *r*_LASSO_ = 0.54), and coffee (*r*_RRR-VIP_ = 0.51; *r*_LASSO_ = 0.51) as estimated from 24-HDR (Table 4). According to the AUC for recent intakes assessed from 24-HDR, the best discrimination between consumers and non-consumers for RRR scores of polyphenol patterns was observed for coffee (AUC_RRR-VIP_ = 89.1%, 95% CI = 82.9–95.4%; AUC_LASSO_ = 89.6%, 95% CI = 83.6–95.6%), followed by red wine (AUC_RRR-VIP_ = 89.1%, 95% CI = 83.5–94.7%; AUC_LASSO_ = 89.1%, 95% CI = 83.6–94.7%), olives (AUC_RRR-VIP_ = 82.2%, 95% CI = 72.9–91.6%; AUC_LASSO_ = 81.0%, 95% CI = 70.9–91.2%), and citrus fruits (AUC_RRR-VIP_ = 81.7%, 95% CI = 76.2–87.2%; AUC_LASSO_ = 81.8%, 95% CI = 76.1–87.5%). When compared with those for single polyphenols, the performance has been improved using polyphenol patterns identified though RRR, especially for coffee (*r* = 0.51, AUC = 89.1% for PPs pattern vs. *r* = 0.42, AUC = 85.8% for single PP) and olives (*r* = 0.35, AUC = 82.2% for PPs pattern vs. *r* = 0.29, AUC = 79.6% for single PP). Correlation coefficients and AUCs for 24-HDR data were consistently higher than those for DQ data. AUCs for habitual dietary intake assessed with DQ were all lower than 80%, except for intake of coffee (*r*_RRR-VIP_ = 0.39; AUC_RRR-VIP_ = 82.7%, 95% CI = 72.2–93.2% and *r*_LASSO_ = 0.42; AUC_LASSO_ = 83.4%, 95% CI = 73.0–93.7% for PPs pattern vs. *r* = 0.38; AUC = 80.9%, 95% CI = 68.9–92.8% for single PP) (Table 4). Table S6 shows the correlation coefficients and AUC for the RRR scores of the polyphenol metabolite patterns in all subjects without applying a cross-validation. These results represent thus a potential upper limit of performance in explaining polyphenol-rich food intake, but cannot be generalized to an independent dataset.

## 4. Discussion

We developed a novel statistical algorithm using a combination of dimension reduction and variable selection methods to integrate high-dimensional biomarker data, with the goal to complement self-reported dietary assessment methods and to improve dietary intake estimation in nutritional epidemiological studies. Here, we applied this approach to a panel of polyphenol metabolites measured in human urine and related dietary intake data. Among 34 targeted urinary polyphenol metabolites, optimal sub-sets were selected by RRR-VIP and LASSO methods, and these patterns of polyphenol metabolites derived by RRR models outperformed any single best polyphenol metabolite associated with the intake of polyphenol-rich foods, especially for coffee and olives.

Polyphenols are widely distributed in plant-based foods such as fruits, vegetables, tea, coffee and wine [11]. A previous study on dietary polyphenol intake in European countries [31] reported an average intake range of total polyphenol of 744–1786 mg/day and 584–1626 mg/day in men and women, respectively, and the main food sources of polyphenols were coffee (21–36%), tea (17–41%), fruits (9–25%), wine (10%) in Mediterranean (MED) countries, non-MED countries and the UK. In this study, polyphenol-rich food groups were pre-selected based on the correlation between food groups and individual polyphenol metabolites prior to the main analyses. Similar to the previous study, fruits (citrus fruits, apples and pears, and olives), coffee, tea, and wine food groups were also selected as polyphenol-rich food groups. Despite vegetables being regarded as a food group rich in polyphenols generally, none of the vegetable sub-groups reached our criteria for being selected as a polyphenol-rich food group. This might be explained by the observation that vegetables overall contributed only less than 5% to polyphenol intake in the EPIC study [31], and different vegetable sub-groups may thus contribute only marginally to polyphenol intake, at least in the EPIC populations.

Recently, individual polyphenol metabolites have been identified as potential biomarkers of dietary polyphenol intake [32,33]. A number of studies examined the potential role of polyphenols as dietary biomarkers in clinical trials or observational studies [17,18,34,35,36,37,38,39,40]. Previous dietary intervention studies [34,35,36,37] have identified that flavonoids such as hesperetin, naringenin, kaempferol, phloretin, and quercetin in 24-h urine could be specific biomarkers for intakes of fruits and vegetables. Other clinical and observational studies [17,38,39,40] found that some 24-h urinary polyphenols were good indicators of polyphenol-rich beverage consumption, such as gallic acids and resveratrol for wine, chlorogenic acid for coffee, and epicatechin for tea. However, all these previous studies examined individual polyphenol metabolites, and to the best of our knowledge, this is the first study using polyphenol metabolite patterns to investigate associations with food intake.

Conceptually similar to dietary pattern analyses [41], free-living people do not consume single polyphenols, but a combination of polyphenols coming from different food sources. Therefore, it is meaningful from a biological point of view to examine combinations of polyphenols, which is also a more comprehensive and efficient approach from a statistical point of view. In this study, we applied dimension reduction and variable selection methods to identify specific urinary polyphenol metabolite patterns associated with the intake of polyphenol-rich foods. RRR analysis is a multivariate dimension reduction technique to determine linear combinations of a set of predictors maximizing the explained variability in responses. RRR has been previously used along with principal component analysis, factor analyses, or cluster analyses for dietary pattern discovery in nutritional epidemiology [42,43]. RRR analysis is similar to partial least squares (PLS) analysis; they are both widely used in analyses of metabolomics data, and both are supervised approaches with regression-based models to reduce dimensions by extracting linear combinations of X-variables that explain variability in Y-variables [44,45]. The difference between RRR and PLS is that RRR focuses on explaining variation in Y-variables, whereas PLS seeks factors whereby the covariance between the X- and the Y-components is maximized. Therefore, in this study, applying the RRR method enabled the identification of patterns of polyphenol metabolites that maximized the explained variability of intakes of specific polyphenol-rich food groups.

For RRR analyses, sub-sets of polyphenol metabolites were pre-selected using variable selection methods: RRR-VIP and LASSO. Previous studies have already observed that most of the selected polyphenols by the two methods here are associated with polyphenol-rich foods or food groups. Hesperetin and naringenin are known abundant polyphenols in citrus fruits [46], and 3,4-dihydroxyphenlylacetic acid (3,4-DHPAA) and 3-hydroxyphenlylacetic acid (3-HPAA) are two metabolites formed from hesperetin and naringenin by the colonic microbiota [47]. Hydroxytyrosol, thyrosol and 3,4-DHPAA are predominant polyphenols in olives [48]. Caffeic acid and chlorogenic acid are two major polyphenols from coffee [49], which are metabolized into 3,4-DHPAA, protocatechuic acid, m-coumaric acid, and vanillic acid by the gut microbiota [50]. Gallic acid ethyl ester and resveratrol are the main polyphenols in red wine [48]. These selected polyphenols included all polyphenols with high coefficients in univariate comparisons. However, selected polyphenol metabolites differed by method, even though the first one or two metabolites were common in both methods. The LASSO method selected more polyphenol metabolites that were negatively associated with polyphenol-rich food intake. It seems that the variable selection using LASSO may be more affected by other factors, such as dietary patterns, while the RRR-VIP method may focus on explaining polyphenol-rich food intake itself. Despite this difference in selected polyphenol metabolites, both methods performed equally well in explaining polyphenol-rich food intake, and both methods were overall equally efficient. Future studies may again compare the performance of both methods in different study settings.

The patterns of urinary polyphenol metabolites in this study better explained acute intake assessed by 24-HDR than habitual intake assessed by DQ, and performed equally bad as any single polyphenol metabolite (Table 4). A review paper [32] suggested that urinary polyphenol metabolites were useful as biomarkers for recent intake (12–72 h) based on results of some clinical trials and on knowledge on their pharmacokinetic properties (median half-life of 2.8 h) [51]. However, relatively high stability over time has been observed for a number of polyphenol metabolites, and this is most likely explained by the frequent consumption of their main food sources [33]. This is what is observed here for coffee and wine, and this also explains the correlations observed with DQ data. For other polyphenol metabolites less frequently consumed, reproducibility in urine may be lower, and this should largely explain the lower correlation of metabolite patterns with DQ data when compared to 24-HDR data. Therefore, polyphenol metabolite patterns could be used as biomarkers for acute dietary intake of polyphenol-containing foods, or for the regular intake of more frequently consumed foods, such as coffee or wine.

The strength of this study includes, first, its statistical design/strategy, which can be easily applied to other “-omics” datasets to identify potential biomarker patterns that can serve as dietary exposure markers. Second, the availability of 24-h urine samples offered additional advantages for the accurate assessment of polyphenol metabolites over spot urine or plasma samples, which are mainly available in most other cohort studies. However, our study had also some limitations. The study design or urinary polyphenol metabolites used in this study did not allow the identification of biomarkers for habitual dietary intake, except for frequently consumed foods (coffee or wine), hence, further research is needed to identify longer-term biomarkers. In addition, this study was carried out in European populations, so the statistical algorithm for identifying polyphenol metabolite patterns should be adapted to other populations as well.

## 5. Conclusions

Urinary polyphenol metabolite patterns performed better or equally well as compared to any single best polyphenol metabolite biomarker for intakes of specific polyphenol-rich foods, especially for acute dietary intake or regular intake of frequently consumed foods. The algorithm developed using dimension reduction and variable selection could be easily extended to other metabolites, foods, and food constituents.

## Figures and Tables

**Table 1:** General characteristics ^a^ of the total study population (*n* = 475).

	Total	Men	Women	*p* ^b^
N (%)	475 (100)	198 (41.7)	277 (58.3)	
Age (years)	53.9 (8.5)	55.4 (8.4)	52.9 (8.4)	0.017
BMI (kg/m^2^)	26.0 (4.3)	26.8 (3.5)	25.5 (4.7)	0.059
Energy intake (kcal/day)	2200.0 (785.5)	2562.7 (830.9)	1940.8 (636.4)	<0.0001
Alcohol intake (g/day)	15.5 (21.1)	23.5 (26.3)	9.7 (13.8)	<0.0001
Smoking status (%)				0.102
Never	50.7	35.9	61.4	
Former	27.2	38.4	19.1	
Current	19.4	23.2	16.6	
Unknown	2.7	2.5	2.9	
Physical activity (%)				0.712
Inactive	26.3	24.8	27.4	
Moderately inactive	40.0	39.9	40.1	
Moderately active	21.3	21.2	21.3	
Active	12.4	14.1	11.2	
Diabetes (%) ^c^	2.5	3.5	1.8	0.213
Hyperlipidemia (%) ^c^	27.2	33.3	22.7	0.087
Hypertension (%) ^c^	23.6	27.8	20.7	0.720

^a^ Mean (SD) or Percentage (%); ^b^ *p*-values for the difference between men and women from the regression—center-adjusted linear regression (continuous variables) or logistic regression (categorical variables); ^c^ Self-reported by questionnaires at recruitment into the study.

**Table 2:** Correlation coefficients ^a^ between urinary polyphenols and intakes of polyphenol-rich foods from 24-HDR among total subjects (*n* = 475).

Polyphenols (*n* = 34)	Food Groups (% Consumers)						
	Citrus Fruits (38.9%)	Apple & Pear (47.6%)	Olives (9.3%)	Coffee (86.3%)	Tea (24.6%)	All Wine (41.9%)	Red Wine (25.5%)
Protocatechuic acid	0.020	0.018	0.055	0.373	−0.116	0.119	0.109
Hydroxytyrosol	0.020	0.010	0.360	0.010	0.100	0.430	0.336
3,5-Dihydroxybenzoic acid	0.080	0.023	0.034	−0.093	0.130	−0.016	−0.027
3,4-Dihydroxyphenylacetic acid	0.174	0.134	0.312	0.028	0.053	0.134	0.116
Genistein	0.076	0.018	−0.027	−0.093	0.067	−0.072	−0.047
Apigenin	0.088	0.055	0.014	−0.062	−0.027	−0.081	−0.064
3,4-Dihydroxyphenylpropionic acid	0.062	0.086	0.012	0.403	−0.159	0.038	0.025
3,5-Dihydroxyphenylpropionic acid	0.077	0.022	0.020	−0.043	0.142	0.050	0.055
3-Hydroxybenzoic acid	0.029	0.024	−0.013	0.162	0.077	0.052	0.091
4-Hydroxybenzoic acid	0.191	−0.031	0.071	0.094	0.008	0.009	0.010
Tyrosol	−0.079	−0.084	0.117	0.045	0.037	0.429	0.317
3-Hydroxyphenylacetic acid	0.121	0.141	0.058	0.027	0.034	0.060	0.063
4-Hydroxyphenylacetic acid	−0.014	−0.060	0.054	0.012	−0.011	0.220	0.164
m-Coumaric acid	0.054	−0.022	0.001	0.294	−0.092	0.113	0.128
p-Coumaric acid	0.011	0.088	0.126	0.104	0.061	0.270	0.212
Vanillic acid	−0.014	0.000	0.009	0.107	−0.065	−0.017	0.024
Naringenin	0.498	0.070	0.064	0.036	−0.018	0.025	−0.043
Phloretin	0.151	0.303	−0.009	0.000	−0.005	−0.027	−0.057
Kaempferol	0.279	0.085	0.036	0.003	0.083	−0.002	−0.021
Epicatechin	0.020	0.233	−0.015	−0.126	0.193	0.135	0.123
Catechin	−0.069	0.003	0.018	−0.098	0.110	0.280	0.280
Hesperetin	0.535	0.056	0.023	0.037	−0.061	0.004	−0.003
Homovanillic acid	0.126	0.117	0.241	−0.081	0.059	0.065	0.069
Isorhamnetin	0.032	0.070	0.036	−0.055	0.074	0.047	0.078
Ferulic acid	0.170	0.053	0.028	0.422	−0.113	0.036	0.003
Resveratrol	0.028	−0.049	0.007	0.012	−0.007	0.409	0.457
Quercetin	0.190	0.083	−0.008	−0.118	0.133	0.126	0.141
Caffeic acid	0.068	0.092	0.049	0.487	−0.121	0.119	0.084
Equol	−0.060	−0.068	−0.040	−0.099	0.049	0.009	0.060
Daidzein	0.043	−0.037	−0.008	−0.115	0.089	−0.023	−0.029
Enterolactone	0.050	0.045	0.105	0.019	0.042	0.077	0.032
Enterodiol	0.067	0.000	0.053	−0.015	0.016	0.018	0.027
Gallic acid	0.055	0.064	0.039	−0.125	0.316	0.344	0.380
Gallic acid ethyl ester	−0.016	−0.030	0.032	−0.009	0.058	0.508	0.654

^a^ Partial Pearson correlation with sex, BMI and age as covariates. Urinary polyphenols were adjusted for center and batch and intakes of food groups were adjusted for energy intake using residuals from general linear models (GLMs). Positive coefficients in blue cells were significant (*p* < 0.05) and higher coefficients had darker color.

**Table 3:** Selected polyphenol metabolites ^a^ by reduced rank regression-based variable importance in projection (RRR-VIP) or least absolute shrinkage and selection operator (LASSO) methods (*n* = 475).

Food Groups	RRR-VIP		LASSO	
	Polyphenol Metabolites	VIP	Polyphenol Metabolites	Coefficients
**Citrus fruits**	Naringenin	2.876	Hesperetin	0.851
	Hesperetin	2.701	Naringenin	0.510
	3,4-Dihydroxyphenylacetic acid	2.552	3,4-Dihydroxyphenylacetic acid	0.091
	Resveratrol	1.207	3-Hydroxyphenylacetic acid	0.086
	3,4-Dihydroxyphenylpropionic acid	1.007	Vanillic acid	−0.009
	m-Coumaric acid	0.970	Apigenin	−0.035
	Genistein	0.927	Tyrosol	−0.037
	Homovanillic acid	0.894	Catechin	−0.046
	Catechin	0.888	4-Hydroxyphenylacetic acid	−0.089
	Daidzein	0.880		
	Hydroxytyrosol	0.855		
**Apples & Pears**	Phloretin	2.666	Phloretin	0.598
	Epicatechin	2.463	Epicatechin	0.199
	Protocatechuic acid	2.047		
	Gallic acid ethyl ester	1.426		
	3,4-Dihydroxyphenylpropionic acid	1.254		
	Enterolactone	1.163		
	Catechin	1.119		
	3,4-Dihydroxyphenylacetic acid	0.914		
	Homovanillic acid	0.913		
	Apigenin	0.889		
	Daidzein	0.883		
**Olives**	Hydroxytyrosol	4.866	Hydroxytyrosol	0.313
	Tyrosol	1.810	3,4-Dihydroxyphenylacetic acid	0.099
	Quercetin	1.382	Catechin	−0.003
	3,4-Dihydroxyphenylacetic acid	0.945	m-Coumaric acid	−0.006
	Gallic acid ethyl ester	0.863	Epicatechin	−0.011
			3-Hydroxybenzoic acid	−0.014
			Gallic acid ethyl ester	−0.028
			Resveratrol	−0.041
			Tyrosol	−0.054
			Quercetin	−0.078
**Coffee**	Caffeic acid	3.559	Caffeic acid	0.853
	Ferulic acid	1.906	Ferulic acid	0.227
	3,4-Dihydroxyphenylacetic acid	1.690	Protocatechuic acid	0.075
	Gallic acid	1.493	3,4-Dihydroxyphenylpropionic acid	0.071
	Apigenin	1.270	Homovanillic acid	−0.013
	Quercetin	1.261	Catechin	−0.016
	Homovanillic acid	1.149	3,5-Dihydroxyphenylpropionic acid	−0.027
	Protocatechuic acid	1.141	4-Hydroxyphenylacetic acid	−0.041
	m-Coumaric acid	1.037	Equol	−0.047
	Hydroxytyrosol	0.879	3,5-Dihydroxybenzoic acid	−0.069
	Daidzein	0.872	Daidzein	−0.076
			Epicatechin	−0.109
			Gallic acid	−0.130
			Apigenin	−0.163
			Quercetin	−0.263
**Tea**	Gallic acid	3.265	Gallic acid	0.977
	Hydroxytyrosol	2.084	3-Hydroxybenzoic acid	0.328
	Protocatechuic acid	1.813	Hydroxytyrosol	0.255
	3,4-Dihydroxyphenylacetic acid	1.520	3,5-Dihydroxyphenylpropionic acid	0.233
	3-Hydroxybenzoic acid	1.462	Kaempferol	0.177
	m-Coumaric acid	1.379	Daidzein	0.140
	3,5-Dihydroxyphenylpropionic acid	0.969	4-Hydroxybenzoic acid	0.072
	Resveratrol	0.959	Genistein	0.057
	Gallic acid ethyl ester	0.857	p-Coumaric acid	0.044
			Epicatechin	0.037
			Quercetin	0.021
			Isorhamnetin	0.018
			Enterodiol	0.006
			Ferulic acid	−0.001
			Apigenin	−0.017
			Tyrosol	−0.037
			3,4-Dihydroxyphenylpropionic acid	−0.109
			3,4-Dihydroxyphenylacetic acid	−0.112
			Gallic acid ethyl ester	−0.119
			3-Hydroxyphenylacetic acid	−0.133
			Phloretin	−0.145
			Hesperetin	−0.148
			4-Hydroxyphenylacetic acid	−0.204
			m-Coumaric acid	−0.260
			Resveratrol	−0.321
			Protocatechuic acid	−0.452
**All wine**	Hydroxytyrosol	3.547	Gallic acid ethyl ester	0.808
	Gallic acid ethyl ester	3.058	Hydroxytyrosol	0.579
	Homovanillic acid	1.531	Tyrosol	0.198
	3-Hydroxybenzoic acid	1.201	Gallic acid	0.068
	Naringenin	1.081	p-Coumaric acid	0.060
	3,4-Dihydroxyphenylpropionic acid	1.010	Enterolactone	0.048
	3,4-Dihydroxyphenylacetic acid	0.909	Catechin	0.023
			Apigenin	−0.063
			3-Hydroxybenzoic acid	−0.089
			Vanillic acid	−0.097
			Homovanillic acid	−0.251
**Red wine**	Gallic acid ethyl ester	5.388	**Gallic acid ethyl ester**	1.333
	Resveratrol	1.315		

^a^ Polyphenols in bold were selected by both RRR-VIP and LASSO methods. The positive (blue) or negative (red) association of selected polyphenols with intakes of food/food groups were shown in different colors.

**Table 4:** Correlations coefficients and area under the receiver operating characteristic curves (ROC AUCs) of RRR scores of selected polyphenol (PP) metabolites with polyphenol-rich foods from 24-HDR and DQ in the test set (*n* = 236).

Food Groups ^a^	Selected PPs ^b^	24-HDR			DQ		
		Consumers (%)	*r* ^c^	ROC AUC ^d^ (95% CI)	Consumers (%)	*r* ^c^	ROC AUC ^d^ (95% CI)
Citrus fruit	Single PP (Hesperetin)	40%	0.538	81.4% (75.9–86.8)	96%	0.124	66.2% (48.8–83.6)
	PPs by RRR-VIP (*n* = 11)		0.543	81.7% (76.2–87.2)		0.139	71.6% (57.9–85.2)
	PPs by LASSO (*n* = 11)		0.539	81.8% (76.1–87.5)		0.163	69.8% (54.9–84.7)
Apples & Pears	Single PP (Phloretin)	48%	0.322	74.2% (68.0–80.5)	96%	0.183	70.6% (54.0–87.2)
	PPs by RRR-VIP (*n* = 10)		0.359	73.5% (67.2–79.8)		0.242	77.7% (61.5–93.9)
	PPs by LASSO (*n* = 2)		0.356	74.3% (68.0–80.6)		0.201	68.5% (51.7–85.3)
Olives	Single PP (Hydroxytyrosol)	8%	0.287	79.6% (69.7–89.5)	26%	0.141	64.8% (56.7–72.9)
	PPs by RRR-VIP (*n* = 5)		0.351	82.2% (72.9–91.6)		0.131	64.1% (55.8–72.4)
	PPs by LASSO (*n* = 10)		0.348	81.0% (70.9–91.2)		0.125	64.2% (56.0–72.5)
Coffee	Single PP (Caffeic acid)	86%	0.416	85.8% (77.7–93.8)	94%	0.383	80.9% (68.9–92.8)
	PPs by RRR-VIP (*n* = 11)		0.505	89.1% (82.9–95.4)		0.392	82.7% (72.2–93.2)
	PPs by LASSO (*n* = 15)		0.510	89.6% (83.6–95.6)		0.417	83.4% (73.0–93.7)
Tea	Single PP (Gallic acid)	25%	0.304	70.5% (62.8–78.2)	64%	0.151	59.8% (52.2–67.5)
	PPs by RRR-VIP (*n* = 9)		0.412	73.9% (66.4–81.4)		0.289	65.0% (57.9–72.1)
	PPs by LASSO (*n* = 26)		0.370	72.4% (65.0–79.8)		0.210	63.2% (55.9–70.5)
All wine	Single PP (Gallic acid ethyl ester)	37%	0.514	76.7% (70.1–83.4)	85%	0.406	74.8% (66.4–83.2)
	PPs by RRR-VIP (*n* = 7)		0.529	77.8% (71.3–84.4)		0.423	76.1% (68.1–84.1)
	PPs by LASSO (*n* = 11)		0.531	77.1% (70.8–83.4)		0.433	76.7% (68.4–84.9)
Red Wine	Single PP (Gallic acid ethyl ester)	23%	0.656	89.1% (83.6–94.7)	24%	0.263	67.8% (59.1–76.4)
	PPs by RRR-VIP (*n* = 2)		0.654	89.1% (83.5–94.7)		0.263	67.8% (59.1–76.4)
	PPs by LASSO (*n* = 1)		0.656	89.1% (83.6–94.7)		0.263	67.8% (59.1–76.4)

^a^ Intakes of food groups were adjusted for energy intake using residuals from general linear models (GLMs); ^b^ Polyphenol metabolites were adjusted for centers and batches using residuals from GLMs; ^c^ Partial Pearson correlation coefficients between RRR scores of selected polyphenols and food groups with sex, BMI and age as covariates; ^d^ ROC AUCs for RRR scores of the patterns of selected polyphenols were calculated and adjusted for sex, BMI and age using logistic regression models.

## Supplementary Materials

The following are available online at www.mdpi.com/2072-6643/9/8/796/s1, Table S1: Correlation coefficients between urinary polyphenols and intakes of whole main food groups from 24-HDR among total subjects (*n* = 475), Table S2: Correlation coefficients between urinary polyphenols and intakes of vegetable groups from 24-HDR among total subjects (*n* = 475). Table S3: Correlation coefficients between urinary polyphenols and intakes of fruit groups from 24-HDR among total subjects (*n* = 475). Table S4: Correlation coefficients between urinary polyphenols and intakes of non-alcoholic beverage groups from 24-HDR among total subjects (*n* = 475). Table S5: Correlation coefficients between urinary polyphenols and intakes of alcoholic beverage groups from 24-HDR among total subjects (*n* = 475). Table S6: Correlations coefficients and ROC AUCs of RRR scores of selected polyphenol (PP) metabolites with polyphenol-rich foods from 24-HDR and DQ in total subjects (*n* = 475).
