# Supplementary material for: Identification of Urinary Polyphenol Metabolite Patterns Associated with Polyphenol-Rich Food Intake in Adults from Four European Countries
Source: Nutrients. 2017 Jul 25;9(8):796. doi: 10.3390/nu9080796 (PMC5579590; doi:10.3390/nu9080796)
Supplement: Supplementary file 1 [file nutrients-09-00796-s001.zip › nutrients-210656-supplementary.pdf]

Table S1. Correlation coefficients[a] between urinary polyphenols and intakes of whole main food groups from 24-HDR among total subjects (n=475).

Polyphenols (n=34)	Food groups (Consumers %)											
	Potatoes (39.8%)	Vegetables (91.8%)	Legumes (8.0%)	Fruit, nuts & seeds (88.2%)	Dairy products (92.4%)	Cereals (99.8%)	Meat & Meat products (82.9%)	Fish and shellfish (26.9%)	Eggs (23.4%)	Fat (95.4%)	Non alcoholic beverages (99.2%)	Alcoholic beverages (57.3%)
Protocatechuic acid	-0.101	0.064	0.075	0.029	0.028	-0.137	0.012	-0.022	0.022	-0.076	0.127	0.070
Hydroxytyrosol	-0.075	0.180	0.042	0.049	-0.094	-0.015	-0.011	0.118	-0.007	-0.001	0.003	0.394
3,5-Dihydroxybenzoic acid	-0.086	0.111	0.026	0.069	0.094	0.193	-0.085	-0.004	-0.077	0.095	0.061	-0.120
3,4-Dihydroxyphenylacetic acid	-0.069	0.120	0.039	0.241	-0.089	-0.026	-0.047	0.056	-0.016	0.032	0.054	0.084
Genistein	0.149	0.025	0.011	0.060	-0.073	-0.046	-0.104	0.025	-0.045	0.044	-0.018	-0.086
Apigenin	-0.004	0.097	0.009	0.059	-0.079	-0.010	-0.002	-0.044	-0.086	0.018	0.024	-0.147
3,4-Dihydroxyphenylpropionic acid	-0.104	-0.107	-0.070	0.049	-0.007	-0.054	0.011	0.038	0.002	-0.053	0.107	0.034
3,5-Dihydroxyphenylpropionic acid	-0.056	0.090	0.028	0.049	0.044	0.140	-0.079	-0.021	-0.010	0.063	0.074	-0.026
3-Hydroxybenzoic acid	-0.020	0.077	-0.011	0.008	0.036	-0.081	-0.085	0.036	0.087	0.012	0.092	0.124
4-Hydroxybenzoic acid	0.008	0.141	0.082	0.189	-0.042	-0.059	-0.011	0.034	0.041	0.012	0.047	0.025
Tyrosol	-0.032	0.103	-0.023	-0.137	-0.123	-0.007	0.005	0.081	-0.043	-0.060	-0.010	0.586
3-Hydroxyphenylacetic acid	-0.010	0.092	0.027	0.182	0.049	-0.029	-0.091	0.063	-0.046	0.000	0.026	0.029
4-Hydroxyphenylacetic acid	-0.019	0.022	-0.007	-0.074	-0.011	-0.016	0.087	-0.010	0.008	-0.121	-0.032	0.356
m-Coumaric acid	-0.008	0.005	-0.034	-0.045	-0.031	-0.100	-0.006	0.041	-0.001	0.013	0.040	0.194
p-Coumaric acid	0.008	0.099	0.002	0.135	-0.087	-0.053	-0.015	0.080	-0.070	-0.072	0.080	0.266
Vanillic acid	-0.046	-0.013	0.097	-0.014	-0.002	-0.175	-0.073	-0.013	-0.065	-0.032	0.082	-0.038
Naringenin	-0.092	0.066	-0.028	0.218	-0.020	0.031	0.034	-0.025	-0.116	-0.052	0.043	-0.007
Phloretin	0.013	0.030	-0.012	0.191	0.055	-0.080	-0.030	-0.018	0.011	-0.067	0.078	-0.025
Kaempferol	-0.042	0.082	-0.041	0.161	-0.052	-0.038	0.008	-0.028	-0.142	-0.023	0.033	-0.014
Epicatechin	-0.016	0.009	-0.051	0.154	-0.072	-0.081	-0.018	-0.051	-0.029	0.036	0.035	0.069
Catechin	-0.023	0.060	-0.015	0.097	-0.029	-0.042	0.062	-0.007	-0.021	0.010	-0.005	0.300
Hesperetin	-0.049	0.012	-0.005	0.229	-0.018	-0.017	0.018	0.009	-0.140	-0.024	0.022	0.030
Homovanillic acid	0.001	0.118	-0.002	0.166	-0.052	0.005	-0.050	0.066	-0.078	0.005	0.081	0.042
Isorhamnetin	0.004	0.070	-0.009	0.114	-0.093	-0.090	-0.055	0.036	-0.098	0.045	0.109	0.000
Ferulic acid	-0.114	-0.015	0.047	0.085	-0.027	-0.006	0.001	-0.031	-0.028	-0.087	0.084	0.083
Resveratrol	0.009	0.052	-0.026	-0.070	-0.016	-0.056	0.081	0.036	-0.046	-0.147	0.001	0.368
Quercetin	0.019	0.141	-0.015	0.113	-0.112	-0.120	0.008	0.036	-0.095	0.062	0.098	0.067
Caffeic acid	-0.107	0.007	-0.010	0.012	0.004	-0.072	0.038	-0.008	-0.012	-0.058	0.105	0.122
Equol	0.146	0.041	-0.030	0.015	0.000	-0.014	-0.107	-0.029	-0.016	0.072	-0.039	0.008
Daidzein	0.103	0.030	0.066	0.049	-0.055	-0.060	-0.134	0.070	-0.002	0.079	-0.022	-0.021
Enterolactone	0.021	0.129	0.050	0.123	0.065	0.118	-0.029	-0.044	0.070	-0.057	0.002	0.102
Enterodiol	0.063	0.048	0.013	0.092	-0.040	0.037	-0.075	-0.036	0.029	-0.046	0.044	-0.013
Gallic acid	-0.072	0.071	0.052	0.010	0.004	-0.045	0.070	-0.034	0.013	-0.006	0.020	0.306
Gallic acid ethyl ester	-0.023	0.065	-0.037	-0.068	-0.019	-0.076	0.091	0.033	-0.007	-0.118	-0.021	0.437

[a] Partial Pearson correlation with sex, BMI and age covariates. Urinary polyphenols were adjusted for center and batch and intakes of food groups were adjusted for energy intake using residuals from General Linear Models (GLMs). Positive coefficients in blue were significant (P<0.05) and higher coefficients had darker color.

Table S2. Correlation coefficients[a] between urinary polyphenols and intakes of vegetable groups from 24-HDR among total subjects (n=475).

Polyphenols (n=34)	Food groups (Consumers %)									
	Vegetables (91.8%)	Leafy vegetables (35.2%)	Fruiting vegetables (62.5%)	Root vegetables (21.3%)	Cabbages (15.4%)	Mushrooms (9.1%)	Grain and pod vegetables (12.0%)	Onion, garlic (46.3%)	Stalk vegetables, sprouts (14.9%)	Mixed salad, mixed vegetables (11.4%)
Protocatechuic acid	0.064	0.066	-0.010	0.027	0.071	0.029	0.015	-0.020	-0.087	0.060
Hydroxytyrosol	0.180	0.075	0.169	0.035	0.044	0.047	0.050	0.016	-0.022	0.133
3,5-Dihydroxybenzoic acid	0.111	0.063	0.131	0.093	-0.045	-0.009	-0.013	0.011	-0.051	0.014
3,4-Dihydroxyphenylacetic acid	0.120	0.056	0.065	0.042	0.039	0.029	0.014	0.071	-0.057	0.108
Genistein	0.025	0.031	-0.034	0.053	0.008	-0.036	0.061	-0.003	0.028	0.005
Apigenin	0.097	0.099	0.052	0.093	0.021	0.023	-0.039	0.085	0.023	0.154
3,4-Dihydroxyphenylpropionic acid	-0.107	0.009	-0.142	0.032	-0.008	0.001	-0.046	-0.063	-0.020	0.012
3,5-Dihydroxyphenylpropionic acid	0.090	0.058	0.022	0.029	0.076	-0.033	-0.003	0.012	-0.015	0.015
3-Hydroxybenzoic acid	0.077	0.013	0.005	0.000	0.116	-0.040	0.025	-0.038	-0.011	0.019
4-Hydroxybenzoic acid	0.141	-0.019	0.145	0.053	0.035	-0.011	0.002	0.053	-0.018	0.083
Tyrosol	0.103	0.066	0.071	0.049	-0.003	0.058	0.087	-0.063	0.021	0.143
3-Hydroxyphenylacetic acid	0.092	0.062	0.078	0.046	-0.015	-0.024	0.125	0.071	-0.008	-0.010
4-Hydroxyphenylacetic acid	0.022	0.019	-0.003	-0.058	-0.027	0.064	0.060	-0.050	-0.002	0.118
m-Coumaric acid	0.005	-0.004	-0.063	-0.009	0.073	-0.015	-0.001	-0.053	0.038	0.040
p-Coumaric acid	0.099	0.047	0.089	0.047	-0.012	-0.057	0.043	0.000	0.009	0.081
Vanillic acid	-0.013	0.050	0.003	0.032	0.022	-0.061	-0.046	-0.003	-0.069	-0.058
Naringenin	0.066	0.058	0.029	-0.045	0.034	0.105	-0.090	0.079	0.052	0.002
Phloretin	0.030	0.022	-0.004	-0.042	0.074	-0.079	-0.012	0.057	-0.045	-0.018
Kaempferol	0.082	0.042	-0.023	-0.012	0.097	0.036	-0.003	0.071	0.080	0.005
Epicatechin	0.009	0.042	-0.053	0.012	0.033	-0.086	0.072	-0.028	-0.041	0.009
Catechin	0.060	0.006	0.029	-0.028	0.064	-0.037	0.082	-0.006	0.048	0.028
Hesperetin	0.012	0.030	-0.058	0.004	0.054	0.132	-0.040	0.055	0.011	-0.007
Homovanillic acid	0.118	0.055	0.052	0.074	-0.002	-0.019	0.060	0.065	-0.037	0.077
Isorhamnetin	0.070	0.029	0.037	0.047	0.065	0.011	0.052	0.097	-0.035	0.092
Ferulic acid	-0.015	0.035	-0.041	0.023	-0.020	0.027	-0.053	-0.040	-0.039	0.022
Resveratrol	0.052	0.000	-0.057	-0.001	0.066	0.028	0.095	-0.074	0.024	0.144
Quercetin	0.141	0.079	0.029	0.023	0.106	-0.020	0.020	0.215	0.007	0.123
Caffeic acid	0.007	0.052	-0.035	0.021	0.046	0.015	-0.014	-0.030	-0.041	0.064
Equol	0.041	0.020	0.066	0.096	0.024	0.002	0.059	0.037	0.056	0.026
Daidzein	0.030	0.011	0.030	0.043	-0.008	-0.044	0.074	0.041	-0.022	0.024
Enterolactone	0.129	0.008	0.102	0.024	0.105	0.008	0.025	0.001	0.028	0.027
Enterodiol	0.048	0.011	-0.002	0.008	0.125	0.011	-0.026	0.047	0.107	-0.001
Gallic acid	0.071	0.064	-0.062	0.037	0.084	-0.026	0.070	0.004	-0.028	0.093
Gallic acid ethyl ester	0.065	0.034	-0.037	-0.027	0.118	0.014	0.118	0.008	-0.030	0.088

[a] Partial Pearson correlation with sex, BMI and age covariates. Urinary polyphenols were adjusted for center and batch and intakes of food groups were adjusted for energy intake using residuals from General Linear Models (GLMs). Positive coefficients in blue were significant ($P<0.05$) and higher coefficients had darker color.

Table S3. Correlation coefficients[a] between urinary polyphenols and intakes of fruit groups from 24-HDR among total subjects (n=475).

Polyphenols (n=34)	Food groups (Consumers %)											
	Fruit (83.8%)	Citrus fruits (38.9%)	Apple and pears (47.6%)	Grape (3.6%)	Stone fruits (16.6%)	Other fruits (9.5%)	Berries (8.8%)	Banana (13.7%)	Kiwi (7.6%)	Nuts & seeds (10.7%)	Mixed fruits (3.8%)	Olives (9.3%)
Protocatechuic acid	0.032	0.020	0.018	-0.012	0.070	0.030	0.051	-0.065	0.048	0.027	-0.020	0.055
Hydroxytyrosol	0.017	0.020	0.010	0.045	0.088	0.004	0.123	-0.064	0.047	0.066	0.021	0.360
3,5-Dihydroxybenzoic acid	0.064	0.080	0.023	-0.005	0.019	-0.006	-0.040	-0.050	-0.026	0.027	-0.027	0.034
3,4-Dihydroxyphenylacetic acid	0.197	0.174	0.134	0.027	0.100	0.025	0.072	-0.010	0.076	0.096	0.008	0.312
Genistein	0.056	0.076	0.018	-0.012	0.072	-0.088	0.004	-0.018	0.017	0.051	-0.063	-0.027
Apigenin	0.040	0.088	0.055	-0.006	0.037	-0.007	0.006	0.006	0.030	0.022	-0.073	0.014
3,4-Dihydroxyphenylpropionic acid	0.073	0.062	0.086	0.008	0.024	0.069	0.023	-0.067	0.047	-0.011	-0.075	0.012
3,5-Dihydroxyphenylpropionic acid	0.037	0.077	0.022	-0.053	0.031	-0.011	-0.037	-0.065	-0.061	0.030	-0.032	0.020
3-Hydroxybenzoic acid	0.023	0.029	0.024	0.029	0.066	-0.070	0.019	-0.072	0.049	-0.068	0.003	-0.013
4-Hydroxybenzoic acid	0.168	0.191	-0.031	-0.021	0.135	0.094	0.058	-0.009	0.041	0.007	-0.026	0.071
Tyrosol	-0.126	-0.079	-0.084	-0.049	0.081	-0.043	0.013	-0.148	-0.010	0.033	0.004	0.117
3-Hydroxyphenylacetic acid	0.166	0.121	0.141	0.027	0.051	-0.031	-0.003	-0.034	0.094	-0.096	0.006	0.058
4-Hydroxyphenylacetic acid	-0.079	-0.014	-0.060	-0.039	0.046	0.006	0.038	-0.057	-0.044	-0.005	-0.003	0.054
m-Coumaric acid	-0.027	0.054	-0.022	-0.059	0.040	-0.017	0.020	-0.067	0.025	-0.014	-0.032	0.001
p-Coumaric acid	0.124	0.011	0.088	-0.076	0.149	0.003	0.212	-0.121	-0.002	0.072	-0.006	0.126
Vanillic acid	-0.026	-0.014	0.000	-0.017	0.022	0.045	-0.017	-0.091	0.012	0.019	-0.001	0.009
Naringenin	0.200	0.498	0.070	0.019	-0.050	-0.008	0.062	0.034	0.018	-0.027	-0.011	0.064
Phloretin	0.194	0.151	0.303	-0.036	-0.046	0.017	-0.060	-0.033	0.065	-0.050	-0.002	-0.009
Kaempferol	0.158	0.279	0.085	-0.034	0.004	-0.021	0.068	0.014	0.021	0.036	-0.028	0.036
Epicatechin	0.156	0.020	0.233	-0.065	0.102	-0.062	-0.006	-0.085	0.049	-0.010	-0.034	-0.015
Catechin	0.107	-0.069	0.003	-0.060	0.246	-0.038	0.303	-0.095	0.063	0.001	-0.025	0.018
Hesperetin	0.198	0.535	0.056	0.023	-0.095	-0.015	-0.053	0.020	0.016	-0.023	0.000	0.023
Homovanillic acid	0.122	0.126	0.117	0.049	0.099	0.026	0.006	-0.031	0.016	0.044	0.029	0.241
Isorhamnetin	0.125	0.032	0.070	-0.012	0.189	-0.050	0.059	-0.059	0.034	-0.001	-0.066	0.036
Ferulic acid	0.102	0.170	0.053	-0.045	0.029	0.048	0.028	-0.055	-0.039	-0.012	-0.057	0.028
Resveratrol	-0.058	0.028	-0.049	0.035	0.020	-0.010	0.043	-0.116	0.009	0.045	-0.003	0.007
Quercetin	0.116	0.190	0.083	-0.039	0.006	-0.050	0.011	-0.013	-0.004	-0.046	-0.055	-0.008
Caffeic acid	0.030	0.068	0.092	-0.011	-0.007	0.014	0.022	-0.086	0.043	-0.010	-0.048	0.049
Equol	0.006	-0.060	-0.068	-0.002	0.081	-0.027	-0.013	0.006	0.032	0.064	-0.021	-0.040
Daidzein	0.036	0.043	-0.037	-0.023	0.047	-0.076	0.043	0.018	0.027	0.003	-0.044	-0.008
Enterolactone	0.093	0.050	0.045	0.045	0.027	0.014	-0.010	0.009	0.044	0.009	0.035	0.105
Enterodiol	0.059	0.067	0.000	0.036	0.013	0.034	-0.017	0.036	0.054	0.021	0.005	0.053
Gallic acid	0.032	0.055	0.064	-0.037	-0.003	-0.033	-0.036	-0.037	0.012	-0.005	-0.084	0.039
Gallic acid ethyl ester	-0.057	-0.016	-0.030	-0.045	0.012	-0.021	0.020	-0.102	0.028	0.007	-0.012	0.032

[a] Partial Pearson correlation with sex, BMI and age as covariates. Urinary polyphenols were adjusted for center and batch and intakes of food groups were adjusted for energy intake using residuals from General Linear Models (GLMs). Positive coefficients in blue were significant (P<0.05) and higher coefficients had darker color.

Table S4. Correlation coefficients[a] between urinary polyphenols and intakes of non-alcoholic beverage groups from 24-HDR among total subjects (n=475).

Polyphenols (n=34)	Food groups (Consumers %)									
	Non alcoholic beverages n.s. (4.4%)	Fruit & vegetable juices (36.2%)	Juice n.s., non citrus juice, mixed juice (11.2%)	Citrus juices (27.6%)	Vegetable juices (0.4%)	Carbonated/soft/iso tonic drinks, diluted syrups (8.6%)	Coffee, tea, herbal teas (96.2%)	Coffee (86.3%)	Tea (24.6%)	Herbal tea (20.0%)
Protocatechuic acid	0.009	0.043	0.051	0.004	0.140	-0.101	0.153	0.373	-0.116	0.041
Hydroxytyrosol	0.026	0.036	-0.021	0.078	0.002	-0.136	0.034	0.010	0.100	0.028
3,5-Dihydroxybenzoic acid	-0.101	0.071	0.079	0.039	0.043	-0.106	0.004	-0.093	0.130	0.139
3,4-Dihydroxyphenylacetic acid	0.052	0.013	0.001	0.014	-0.012	-0.066	0.058	0.028	0.053	0.063
Genistein	-0.020	0.007	-0.017	0.042	0.044	0.045	-0.040	-0.093	0.067	0.086
Apigenin	-0.001	0.038	-0.038	0.074	0.036	-0.069	0.010	-0.062	-0.027	0.205
3,4-Dihydroxyphenylpropionic acid	-0.027	-0.045	-0.024	-0.040	0.081	-0.053	0.175	0.403	-0.159	-0.019
3,5-Dihydroxyphenylpropionic acid	-0.059	0.060	0.026	0.066	0.051	-0.069	0.049	-0.043	0.142	0.101
3-Hydroxybenzoic acid	-0.050	-0.028	-0.005	-0.032	0.146	-0.098	0.116	0.162	0.077	0.023
4-Hydroxybenzoic acid	-0.012	0.034	0.024	0.030	0.026	-0.020	0.048	0.094	0.008	0.010
Tyrosol	0.019	0.038	-0.053	0.104	0.016	-0.132	-0.016	0.045	0.037	-0.114
3-Hydroxyphenylacetic acid	-0.003	0.028	0.025	0.023	0.053	-0.009	0.035	0.027	0.034	0.080
4-Hydroxyphenylacetic acid	0.033	0.065	-0.030	0.134	0.025	-0.092	-0.094	0.012	-0.011	-0.113
m-Coumaric acid	-0.085	-0.025	-0.060	0.019	0.167	-0.047	0.102	0.294	-0.092	-0.056
p-Coumaric acid	0.046	0.083	0.078	0.037	0.066	-0.084	0.101	0.104	0.061	-0.009
Vanillic acid	0.050	0.021	-0.039	0.079	-0.005	-0.081	0.053	0.107	-0.065	0.095
Naringenin	-0.016	0.225	0.077	0.224	0.015	-0.064	0.033	0.036	-0.018	0.003
Phloretin	-0.012	0.064	0.164	-0.069	-0.044	-0.108	-0.002	0.000	-0.005	0.032
Kaempferol	0.008	0.173	0.088	0.140	0.028	-0.094	0.027	0.003	0.083	0.005
Epicatechin	0.010	-0.035	-0.017	-0.035	-0.068	-0.084	-0.007	-0.126	0.193	0.011
Catechin	-0.003	-0.033	-0.056	0.009	-0.042	-0.107	-0.044	-0.098	0.110	-0.052
Hesperetin	-0.052	0.185	0.034	0.203	0.012	-0.026	0.008	0.037	-0.061	0.015
Homovanillic acid	0.049	0.016	-0.001	0.026	-0.017	-0.145	0.023	-0.081	0.059	0.108
Isorhamnetin	0.034	-0.077	-0.065	-0.033	-0.004	-0.128	0.068	-0.055	0.074	0.162
Ferulic acid	-0.023	0.052	-0.029	0.101	0.082	-0.106	0.179	0.422	-0.113	-0.082
Resveratrol	0.013	-0.001	0.025	-0.015	-0.003	-0.096	-0.010	0.012	-0.007	-0.035
Quercetin	0.122	0.001	-0.015	0.017	-0.013	-0.101	0.049	-0.118	0.133	0.130
Caffeic acid	-0.082	0.011	0.012	0.003	0.064	-0.080	0.186	0.487	-0.121	-0.058
Equol	-0.030	-0.046	-0.023	-0.040	-0.013	-0.036	-0.004	-0.099	0.049	0.083
Daidzein	-0.040	0.011	-0.042	0.074	0.086	0.042	-0.044	-0.115	0.089	0.082
Enterolactone	-0.046	0.102	0.088	0.056	0.019	0.003	0.034	0.019	0.042	0.075
Enterodiol	-0.009	0.035	0.069	0.003	0.016	-0.006	0.025	-0.015	0.016	0.086
Gallic acid	0.006	-0.042	-0.051	-0.010	-0.019	-0.152	0.029	-0.125	0.316	-0.049
Gallic acid ethyl ester	0.033	-0.042	-0.016	-0.015	-0.012	-0.111	-0.025	-0.009	0.058	-0.033

[a] Partial Pearson correlation with sex, BMI and age as covariates. Urinary polyphenols were adjusted for center and batch and intakes of food groups were adjusted for energy intake using residuals from General Linear Models (GLMs). Positive coefficients in blue were significant ($P<0.05$) and higher coefficients had darker color.

Table S5. Correlation coefficients[a] between urinary polyphenols and intakes of alcoholic beverage groups from 24-HDR among total subjects (n=475).

Polyphenols (n=34)	Food groups (Consumers %)											
	Alcoholic beverages (57.3%)	All wine (41.9%)	Red wine (25.5%)	White wine (16.6%)	Rose wine (2.9%)	Beer, cider (14.1%)	Beer (13.1%)	Cider (0.6%)	Spirits, brandy (5.3%)	Aniseed drinks (pastis …) (1.1%)	Liqueurs (3.8%)	Cocktails, punches (2.3%)
Protocatechuic acid	0.070	0.119	0.109	0.051	0.014	-0.066	-0.068	0.040	0.037	-0.016	0.030	0.020
Hydroxytyrosol	0.394	0.430	0.336	0.259	0.068	-0.032	-0.023	-0.031	0.094	0.086	0.094	0.152
3,5-Dihydroxybenzoic acid	-0.120	-0.016	-0.027	0.015	0.000	-0.142	-0.146	0.045	-0.053	-0.016	-0.021	-0.052
3,4-Dihydroxyphenylacetic acid	0.084	0.134	0.116	0.069	0.044	-0.053	-0.045	-0.052	-0.049	0.008	0.126	-0.032
Genistein	-0.086	-0.072	-0.047	-0.025	-0.052	-0.028	-0.019	-0.033	-0.013	-0.002	0.025	-0.130
Apigenin	-0.147	-0.081	-0.064	-0.059	-0.009	-0.055	-0.060	0.017	-0.074	-0.027	-0.076	-0.062
3,4-Dihydroxyphenylpropionic acid	0.034	0.038	0.025	0.020	0.003	0.005	0.008	0.019	0.049	-0.046	0.003	-0.034
3,5-Dihydroxyphenylpropionic acid	-0.026	0.050	0.055	0.015	0.002	-0.115	-0.112	0.002	-0.057	0.010	0.007	-0.029
3-Hydroxybenzoic acid	0.124	0.052	0.091	0.016	-0.039	0.083	0.086	0.006	0.038	-0.061	0.009	-0.013
4-Hydroxybenzoic acid	0.025	0.009	0.010	0.025	-0.038	0.048	0.022	0.060	0.090	0.114	-0.029	-0.035
Tyrosol	0.586	0.429	0.317	0.232	0.148	0.220	0.209	0.079	0.169	0.104	0.057	0.199
3-Hydroxyphenylacetic acid	0.029	0.060	0.063	0.028	-0.001	-0.015	-0.031	0.048	0.046	0.040	0.022	-0.100
4-Hydroxyphenylacetic acid	0.356	0.220	0.164	0.136	0.014	0.189	0.195	0.028	0.045	0.041	0.098	0.088
m-Coumaric acid	0.194	0.113	0.128	0.008	0.008	0.072	0.088	-0.016	0.064	-0.035	0.043	0.075
p-Coumaric acid	0.266	0.270	0.212	0.163	0.019	-0.010	-0.026	0.049	0.173	0.294	-0.001	0.082
Vanillic acid	-0.038	-0.017	0.024	-0.039	-0.023	-0.037	-0.032	-0.015	-0.038	-0.062	0.015	0.009
Naringenin	-0.007	0.025	-0.043	0.077	-0.005	-0.026	-0.031	-0.009	0.024	0.054	-0.065	0.032
Phloretin	-0.025	-0.027	-0.057	-0.018	0.005	0.015	0.004	0.088	-0.020	-0.013	-0.050	0.005
Kaempferol	-0.014	-0.002	-0.021	0.002	-0.001	-0.021	-0.020	-0.015	0.004	0.016	-0.036	0.039
Epicatechin	0.069	0.135	0.123	-0.010	0.107	-0.043	-0.041	-0.038	-0.042	-0.081	0.056	-0.056
Catechin	0.300	0.280	0.280	0.073	0.067	0.086	0.072	-0.049	0.009	-0.009	0.141	0.024
Hesperetin	0.030	0.004	-0.003	0.013	0.013	0.044	0.047	0.028	0.015	0.026	-0.074	0.037
Homovanillic acid	0.042	0.065	0.069	0.010	0.036	-0.028	-0.012	-0.061	-0.045	0.010	0.092	-0.044
Isorhamnetin	0.000	0.047	0.078	-0.057	0.021	-0.029	-0.037	0.048	-0.039	-0.052	0.029	-0.035
Ferulic acid	0.083	0.036	0.003	0.056	0.003	0.048	0.051	-0.002	0.049	-0.020	-0.035	0.044
Resveratrol	0.368	0.409	0.457	0.029	0.128	-0.060	-0.055	-0.013	0.032	0.023	0.062	0.098
Quercetin	0.067	0.126	0.141	0.012	0.046	-0.044	-0.038	-0.020	-0.010	-0.010	0.009	-0.009
Caffeic acid	0.122	0.119	0.084	0.074	0.023	0.016	0.000	0.051	0.107	-0.028	0.010	0.003
Equol	0.008	0.009	0.060	-0.079	-0.038	0.004	0.005	-0.004	-0.039	-0.022	-0.009	-0.095
Daidzein	-0.021	-0.023	-0.029	-0.011	-0.011	0.009	-0.001	0.021	-0.016	0.008	0.052	-0.129
Enterolactone	0.102	0.077	0.032	0.097	-0.025	0.040	0.043	0.018	0.063	0.026	0.028	-0.029
Enterodiol	-0.013	0.018	0.027	0.011	-0.041	0.001	0.009	-0.035	-0.066	-0.055	0.026	-0.090
Gallic acid	0.306	0.344	0.380	0.046	0.085	-0.028	-0.017	-0.032	0.024	-0.024	0.066	0.025
Gallic acid ethyl ester	0.437	0.508	0.654	0.001	0.039	-0.073	-0.067	-0.019	0.011	0.020	0.121	0.095

[a] Partial Pearson correlation with sex, BMI and age. Urinary polyphenols were adjusted for center and batch and intakes of food groups were adjusted for energy intake using residual from General Linear Model (GLM). Positive coefficients in blue were significant (P<0.05) and higher coefficients had darker color.

Table S6. Correlations coefficients and area under the receiver operating characteristic curves (ROC AUCs) of RRR scores of selected polyphenol (PP) metabolites with polyphenol-rich foods from 24-HDR and DQ in total subjects (n=475)

Food groups[a]	Selected PPs[b]	24-HDR					DQ				
		Consumers (%)	r[c]	ROC AUC[d]	Lower 95% CI	Upper 95% CI	Consumers (%)	r[c]	ROC AUC[d]	Lower 95% CI	Upper 95% CI
Citrus fruit	Single PP (Hesperetin)	39%	0.535	81.3%	77.3%	85.3%	97%	0.119	76.0%	64.9%	87.2%
	PPs by RRR-VIP (n=11)		0.570	83.9%	80.2%	87.7%		0.108	75.6%	65.1%	86.2%
	PPs by Lasso (n=11)		0.599	83.7%	79.8%	87.5%		0.143	75.8%	65.0%	86.7%
Apple & Pear	Single PP (Phloretin)	48%	0.303	72.0%	67.4%	76.5%	97%	0.119	67.4%	54.8%	80.0%
	PPs by RRR-VIP (n=10)		0.381	74.2%	69.8%	69.8%		0.167	68.1%	55.8%	80.5%
	PPs by Lasso (n=2)		0.331	72.4%	72.4%	76.9%		0.148	67.6%	55.0%	80.3%
Olives	Single PP (Hydroxytyrosol)	9%	0.360	81.4%	74.8%	88.0%	22%	0.099	61.1%	55.1%	67.2%
	PPs by RRR-VIP (n=5)		0.439	86.6%	81.6%	91.7%		0.085	60.7%	54.6%	66.9%
	PPs by Lasso (n=10)		0.447	86.5%	81.2%	91.8%		0.088	60.8%	54.6%	66.9%
Coffee	Single PP (Caffeic acid)	86%	0.487	86.0%	80.7%	91.2%	94%	0.412	75.1%	65.6%	84.5%
	PPs by RRR-VIP (n=11)		0.591	90.6%	86.5%	94.7%		0.479	80.9%	72.7%	89.1%
	PPs by Lasso (n=15)		0.602	91.4%	87.7%	95.1%		0.480	79.4%	70.9%	87.8%
Tea	Single PP (Gallic acid)	25%	0.316	69.5%	63.8%	75.2%	66%	0.156	58.4%	52.9%	63.8%
	PPs by RRR-VIP (n=9)		0.446	75.8%	70.4%	81.1%		0.263	60.8%	55.6%	65.9%
	PPs by Lasso (n=26)		0.496	79.2%	74.3%	84.1%		0.267	60.4%	55.1%	65.7%
Wine	Single PP (Gallic acid ethyl ester)	42%	0.508	76.6%	72.0%	81.2%	85%	0.407	74.1%	68.0%	80.2%
	PPs by RRR-VIP (n=7)		0.567	80.1%	75.9%	84.2%		0.448	75.5%	69.8%	81.2%
	PPs by Lasso (n=11)		0.579	80.6%	76.6%	84.6%		0.467	77.2%	71.5%	82.9%
Red Wine	Single PP (Gallic acid ethyl ester)	25%	0.654	88.6%	84.8%	92.4%	27%	0.231	64.6%	58.8%	70.5%
	PPs by RRR-VIP (n=2)		0.661	88.3%	84.6%	92.0%		0.225	64.6%	58.8%	70.4%
	PPs by Lasso (n=1)		0.654	88.6%	84.8%	92.4%		0.231	64.6%	58.8%	70.5%

[a] Intakes of food groups were adjusted for energy intake using residuals from General linear models (GLMs).
[b] Polyphenol metabolites were adjusted for centers and batches using residuals from GLMs.
[c] Partial Pearson correlation coefficients between RRR scores of selected polyphenols and food groups with sex, BMI and age covariates.
[d] ROC AUCs for RRR scores of the patterns of selected polyphenols were calculated and adjusted for sex, BMI and age using logistic regression models.
